# Transient TKI-resistant CD44+pBAD+ blasts undergo intrinsic homeostatic adaptation to promote the survival of acute myeloid leukemia *in vitro*

**DOI:** 10.3389/fonc.2023.1286863

**Published:** 2023-11-08

**Authors:** Yi Xu, David J. Baylink, Chien-Shing Chen, Laren Tan, Jeffrey Xiao, Brandon Park, Ismael Valladares, Mark E. Reeves, Huynh Cao

**Affiliations:** ^1^ Division of Hematology and Oncology, Loma Linda University Medical Center and Loma Linda University Cancer Center, Loma Linda University Health, Loma Linda, CA, United States; ^2^ Division of Regenerative Medicine, Department of Medicine, Loma Linda University, Loma Linda, CA, United States; ^3^ Department of Medicine, Loma Linda University School of Medicine, Loma Linda University Health, Loma Linda, CA, United States; ^4^ Department of Pulmonary, Critical Care, Hyperbaric and Sleep Medicine, Loma Linda University Medical Center, Loma Linda, CA, United States

**Keywords:** AML, tyrosine kinase inhibitor, compensation, CD44, JAK/STAT, BAD, Bcl-2, homeostasis

## Abstract

Acute myeloid leukemia (AML) patients have frequent mutations in FMS-like receptor tyrosine kinase 3 (FLT3-mut AML), who respond poorly to salvage chemotherapies and targeted therapies such as tyrosine kinase inhibitors (TKIs). Disease relapse is a common reason of treatment failures in FLT3-mut AML patients, but its intracellular refractory mechanism remains to be discovered. In this study, we designed serial *in vitro* time-course studies to investigate the biomarkers of TKI-resistant blasts and their survival mechanism. First, we found that a group of transient TKI-resistant blasts were CD44+Phosphorylated-BAD (pBAD)+ and that they could initiate the regrowth of blast clusters *in vitro*. Notably, TKI-treatments upregulated the compensation pathways to promote PIM2/3-mediated phosphorylation of BAD to initiate the blast survival. Next, we discovered a novel process of intracellular adaptive responses in these transient TKI-resistant blasts, including upregulated JAK/STAT signaling pathways for PIM2/3 expressions and activated SOCS1/SOCS3/PIAS2 inhibitory pathways to down-regulate redundant signal transduction and kinase phosphorylation to regain intracellular homeostasis. Finally, we found that the combination of TKIs with TYK2/STAT4 pathways-driven inhibitors could effectively treat FLT3-mut AML *in vitro*. In summary, our findings reveal that TKI-treatment can activate a JAK/STAT-PIM2/3 axis-mediated signaling pathways to promote the survival of CD44+pBAD+blasts *in vitro*. Disrupting these TKIs-activated redundant pathways and blast homeostasis could be a novel therapeutic strategy to treat FLT3-mut AML and prevent disease relapse *in vivo*.

## Introduction

Acute Myeloid Leukemia (AML) is a heterogeneous bone marrow (BM) malignancy with some of the lowest survival rates among the leukemias (1, 2). ~35% of AML patients carry oncogenic mutations of FMS-like receptor tyrosine kinase 3 (FLT3) through either an internal tandem duplication (ITD) or a point mutation in the TK domain (TKD) of FLT3 (3). Constitutive activation of mutated FLT3 promotes clonal proliferation through multiple downstream signaling pathways like JAK/STAT5 (4, 5), leading to frequent relapse and a very poor prognosis for AML patients (6).

Over the last few years, multiple tyrosine kinase inhibitors (TKIs), including Midostaurin (MIDO), Sorafenib (SORA), Gilteritinib (GILT), and Quizartinib (QUIZ), have been developed to treat AML with FLT3 mutations (FLT3-mut AML), some of which are in clinical trials and MIDO and GILT have received U.S. Food and Drug Administration (FDA) approval (7, 8). However, due to the clonal heterogeneity of AML, these TKI drugs are typically combined with standard treatments to show improved clinical benefits (9). Earlier studies suggested that acquired mutations of FLT3 receptors could lead to TKI-resistance (10). Furthermore, to support the relapse of FLT3-mut AML, TKI-resistant blasts activate several pro-survival genes through known or unknown signaling pathways (11). Finally, single-cell transcriptomics revealed the complexity of clinical resistance to TKIs (12, 13), suggesting the urgent need to identify specific biomarkers for TKI-resistant blasts in order to develop more effective targeted therapies to treat FLT3-mut AML and prevent relapse (14).

AML is known to have disturbed homeostatic balance between leukemic cell survival and cell death (15). However, the death programs of cancer cells can trigger known and unknown compensatory pathways to reverse the death-signaling at the early stage of apoptosis, leading to the survival of injured blasts (16, 17). The BCL-2 associated agonist of cell death (BAD) protein is a member of BCl-2 gene family that, when de-phosphorylated, can bind to antiapoptotic proteins such as BCL-2 and BCL-XL and promote cell death (18). BAD can also be phosphorylated through classical signaling pathways including AKT, PKA, etc. and perform pro-survival functions involved in cancer development and treatment-resistance in solid tumors (19, 20). Although, accumulating evidence suggests that BAD phosphorylation could be a therapeutic target in oncology, its functional role in AML is not completely understood (21).

In this study, we designed multiple-layer approaches and serial *in vitro* time-course studies through flow cytometry, qPCR and western blot to fulfill the unmet needs in the field by achieving a better understanding of the survival mechanism of TKI-resistant blasts, including 1) identifying novel molecular and genetic biomarkers of TKI-resistant blasts; 2) discovering the process of homeostatic adaptation (defined as intracellular adaptive responses after TKI-treatment) inside survived blasts; 3) exploring the combination of TKIs and pathway-driven small molecule inhibitors to overcome drug resistance *in vitro*.

## Materials and methods

The list of reagents, including manufacturers and catalogs of antibodies, primers, and kits, are found in the supplementary data (**Supplementary Tables 1**, **2**). Biological replicates (N=3) were performed for all experiments including phenotypes of resistance developments at different time intervals and time-course studies of molecular characterizations. Figure results were from one of the three independent experiments (N=3).

### Cell lines and cell culture

MV4-11 (ATCC CRL-9591) is a human-derived AML blast cell line with FLT3-ITD. The AML cells were cultured in RPMI-1640 medium (Hyclone, Thermo Scientific), supplemented with 10% heat-inactivated fetal bovine serum (FBS, HyClone), 1% penicillin/streptomycin. Cells were grown at 37°C in a humidified atmosphere containing 5% CO_2_.

### Small molecular inhibitors and *in vitro* treatment

The list of inhibitors, abbreviations, manufacturers, and catalog # are found in the **supplementary data** (**Supplementary Table 1**). Midostaurin (MIDO) and Sorafenib (SORA) are first-generation FLT3 inhibitors, and Gilteritinib (GILT) and Quizartinib (QUIZ) are second-generation inhibitors (7). As single agents to treat blasts *in vitro*, a single dose of 80nM of MIDO, GILT, QUIZ, or SORA were added to 1 ml of 1x 10^6^ cells for each experimental group in 24 well plates. The dose of 80nM for the four TKIs was selected based on their dose-dependent cytotoxicity in previous reports such as for MIDO (11). Both GILT and QUIZ were reported to start to suppress the sphere-like leukemic clustering and c-Kit at the dose of 80nM (22). The half-life of TKI was previously reported for MIDO (~20 hours) (23), SORA (20-48 hours) (24), GILT (~113 hours) (25) with sustained high-level FLT3 inhibition (26), and QUIZ (~3.5 days, but its active metabolite with prolonged activity up to 14 days *in vitro* after cessation of dosing) (27). As combination agents to treat blasts *in vitro*, one dose of 80nM Gilteritinib with one dose of either 100nM Venetoclax (BCL2-Inhibitor (BCL2-I)), 10µM (R)-Lisofylline (STAT4-I), 100nM AZD1208 (PIM-I), 500nM PF-06826647 (TYK2-I), or 500nM AT9283 (JAK2/3-I) were added to 1 ml of 1x 10^6^ cells for each experimental group in 24 well plates. Three days after the treatment, cells were then collected for either analyses by flow cytometry (FC), qPCR and Western blot (WB), or re-plated into 24-well plates for continued culture (without further TKI treatment) for up to 1 month. We performed medium change every 2-3 days for those continued cell cultures of TKI-treated MV4-11 (one-time TKI-treatment: single dose for first 3 days only). At different time points, re-plated cells were collected for analyses by FC, qPCR and WB.

### Flow cytometry

Cells were harvested and examined for the expression of cell surface biomarkers (CD) and intracellular proteins by multichromatic FC as previously described (28). The viability dye used in this study is Fixable Viability Dye eFluor™ 780 (eBioscience Cat#: 65-0865-14), which can be washed, fixed, permeabilized, and stained for intracellular antigens. After the staining of viability dye, about 1 x 10^4^ ~ 10^6^ cells in 100 µl FC buffer (PBS containing 1% FBS and 0.05% sodium azide) were stained with various fluorescence-conjugated antibodies specific for the desired cell surface proteins at 4°C for 30min. The surface-stained cells were then fixed and permeabilized using the appropriate reagents (e.g. the BD Pharmingen Cytofix/Cytoperm buffer) and stained with different fluorescence-conjugated antibodies (Abs) specific for the desired intracellular proteins at 4°C for 2 hours in the permeabilizing buffer (e.g. the BD Perm/Wash buffer). Concentrations of the Abs were used per the manufacturers’ recommendations. Finally, the cells were washed twice in the permeabilizing buffer and twice in the FC buffer before being analyzed on the BD FACSAria II. Data was analyzed using the FlowJo software (Tree Star Inc., Ashland, OR).

### RNA isolation and real-time polymerase chain reaction analysis

MV4-11 blasts of different experimental groups were collected for RNA isolation and qPCR analysis as previously described (29). Total RNA was isolated using the RNeasy Micro Kit (Qiagen) according to the manufacturer’s instructions. First-strand cDNA was synthesized using the SuperScript III Reverse Transcriptase (Invitrogen; Life Technologies). With an Applied Biosystems 7900HT Real-Time PCR machine, qPCR was performed and analyzed. Primers used in this study are available in **Supplementary Table 2**. The PCR conditions were 10 minutes at 95°C followed by 40 cycles of 10 seconds at 95°C and 30 seconds at 60°C. The relative expression level of a gene was determined using the ΔΔCt method and normalized to GAPDH.

### Western blot analysis

MV4-11 blasts of different experimental groups were collected for western blot analysis as previously described (30). Briefly, equal quantities of protein samples from different treatment groups were loaded onto 10% SDS-polyacrylamide gel and separated by electrophoresis at 100 V for 1 hour. Proteins were then transferred onto Immobilon-P membranes (Millipore Corporation, Billerica, MA), probed with primary antibodies of PIM2 (CST-4730) and PIM3 (CST-4165) with HRP-conjugated secondary antibodies. The exposed films were then developed within minutes, and results were acquired using Kodak ID image software (Eastman Kodak, Rochester, NY).

### Imaging acquisition

Phase-bright images were taken using an Olympus 1X71 inverted microscope and were processed using an Olympus cellSens Dimension 1.15 Imaging Software.

### Statistical analysis

Statistical analyses were performed with GraphPad software (Prism 5.02). The quantitative analyses were analyzed using 1- or 2-way ANOVA followed by a Dunnett’s multiple comparisons test or a Bonferroni *post hoc* analysis as appropriate, or an unpaired *t*-test. All values were presented as mean ± SEM. Results were considered statistically significant when the p-value was <0.05.

## Results

### CD44+pBAD+ blasts survived against TKI treatments *in vitro*

**Figure 1:**
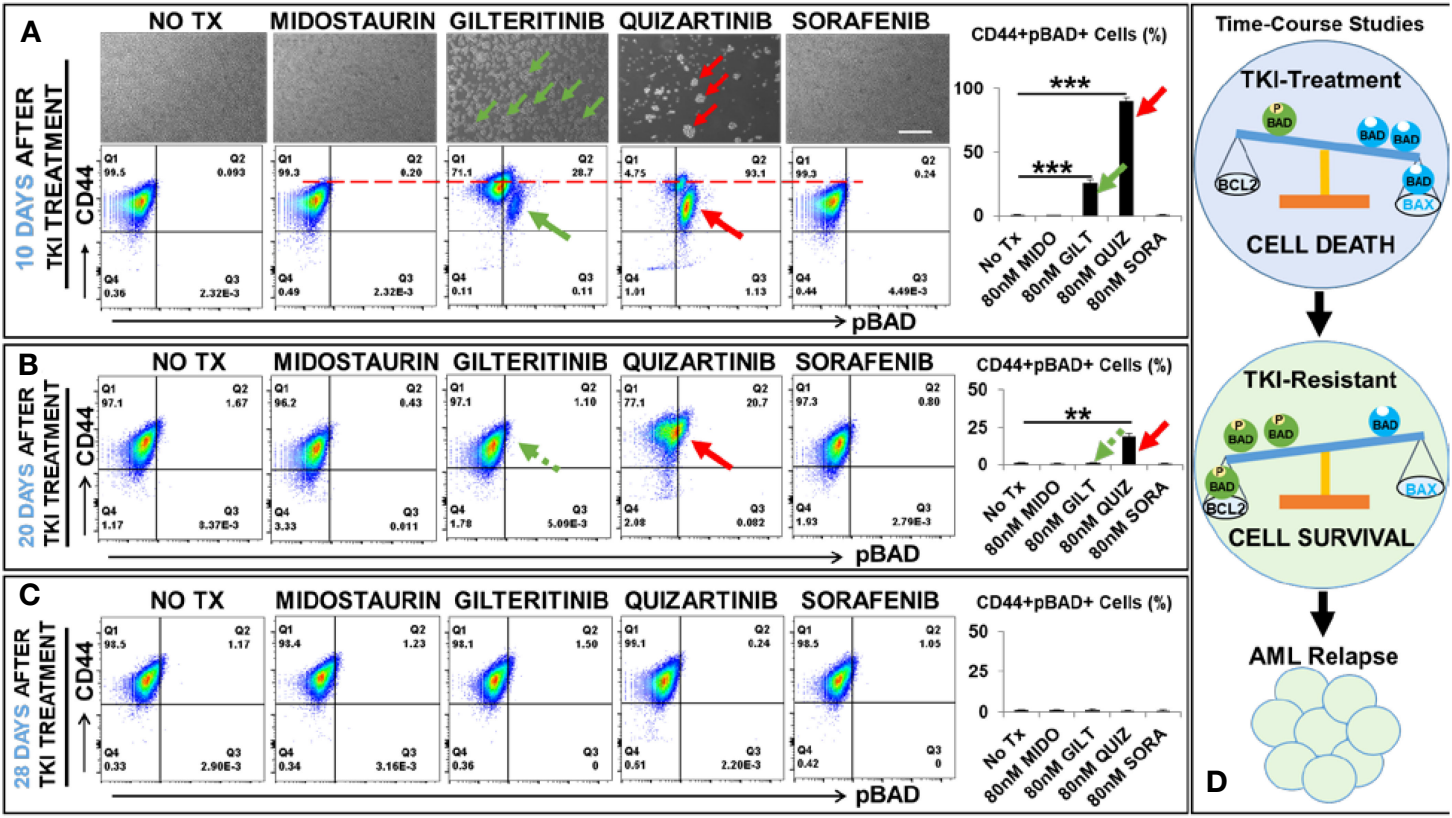
Time-course studies of TKI-resistant CD44+pBAD+ MV4-11 *in vitro.* 10, 20, and 28 days after TKI-treatment *in vitro*, MV4-11 cells of different experimental groups were collected for flow cytometry (FC) analyses as described in Materials and Methods. **(A)** Upper panels: Representative phase-bright images of different treatment groups on day 10 after TKI treatment *in vitro;* Scale bar: 500 µm; Green arrows indicate re-grown clusters in Gilteritinb (GILT)-treated MV4-11 cells; Red arrows indicate re-grown clusters in Quizartinib (QUIZ)-treated MV4-11 cells; Lower panels: Representative FC plots of pBAD expression in different TKI-treatment groups on day 10 after TKI-treatment *in vitro*; Red dash line indicates the CD44high expression in different TKI-treatment groups; Right panel: Cumulative FC percentage data of viable CD44+pBAD+ MV4-11 cells; Green arrows indicate CD44+pBAD+cells in GILT-treated experimental group; Red arrows indicate CD44+pBAD+cells in QUIZ-treated experimental group; **(B)** Representative FC plots of pBAD expression in different TKI-treatment groups on day 20 after TKI-treatment *in vitro*; Right panel: Cumulative FC percentage data of viable CD44+pBAD+ MV4-11 cells; Green arrows indicate CD44+pBAD+cells in GILT-treated experimental group; Red arrows indicate CD44+pBAD+cells in QUIZ-treated experimental group; **(C)** Representative FC plots of pBAD expression in different TKI-treatment groups on day 28 after TKI-treatment *in vitro*; Right panel: Cumulative FC percentage data of viable CD44+pBAD+ MV4-11 cells; **(D)** Schematic diagram of time-course studies illustrating the survival mechanism of TKI-resistant blasts *in vitro*; Where applicable, data are means ± SEM and were analyzed by student “t” test. The significance of each experimental group was based on the NO-TX control group. **P<0.01, ***P<0.005, N=3.

To illustrate the mechanism responsible for the relapse of FLT3-mut AML *in vitro* and identify the potential biomarkers of TKI-resistant blasts, we performed detailed analyses of TKI-treated blasts at different time intervals (3 days, 10 days, 20 days, and 28 days) after exposure to TKIs. First, we performed *in vitro* TKI treatment of MV4-11 blasts with Midostaurin (MIDO), Sorafenib (SORA), Gilteritinib (GILT), and Quizartinib (QUIZ). At different time intervals, microscopy and flow cytometry (FC) were performed to examine morphological changes of TKI-treated blasts, their viability, and alterations of surface biomarkers and intracellular proteins to understand their conversion between life and death (**Figure 1**). As expected, all four TKIs significantly reduced viable MV4-11 when compared to the non-treatment controls after 3-day treatment; however, as the second-generation TKIs, GILT and QUIZ had superior efficacy in promoting cell death and suppressing blast proliferation (Ki67+) when compared to the first-generation FLT3 inhibitors MIDO and SORA (**Supplementary Figure 1**). To follow the cell fate of TKI-treated MV4-11 blasts and characterize their molecular changes over a longer time, we continued to culture blasts by performing medium change every 2-3 days. After 10 days after TKI treatment, we found that culture of both MIDO- and SORA-treated MV4-11 recovered and displayed normal morphologies like the non-treatment controls (images of MIDO and SORA, **Figure 1A**). In contrast, there were many new clusters visualizable among numerous floating dead cells (detectable by trypan blue that confirmed by viability dye through flow cytometry) in the GILT-treated blast culture, and fewer new clusters in the QUIZ-treated blast culture (indicated by green and red arrows respectively in images of GILT and QUIZ, **Figure 1A**). The BCL-2 associated agonist of cell death protein (BAD), a member of the BCL-2 family, is involved in initiating apoptosis since de-phosphorylated BAD is pro-apoptotic by binding BCL-2 and inactivating the pro-survival function of BCL-2 while phosphorylated BAD (pBAD) is anti-apoptotic by leaving BCL-2 available to block the process of BAX-triggered apoptosis (18). To characterize these blasts that survived the TKI-treatment, we performed flow cytometry (FC) analyses. Our FC data revealed significantly increased populations of viable CD44+pBAD+ cells in GILT- (28.7%) and QUIZ- (93.1%) treated blast cultures, in contrast to the non-treatment control (0.093%), MIDO- (0.2%) and SORA- (0.24%) experimental groups (indicated by green and red arrows respectively in FC plots of GILT and QUIZ, **Figure 1A**). Interestingly, CD44+ expression was also increased in GILT- and QUIZ-treated blast cultures at this time-point, when compared to the control and other treatment groups (indicated by the red dash line in FC plots of **Figure 1A**).

After 20 days after TKI treatment, we found that culture of GILT-treated MV4-11 recovered like non-treatment controls. Our FC analyses confirmed that the percentage of viable CD44+pBAD+ cells in GILT-treated group, had decreased to 1.1% (indicated by a green arrow, **Figure 1B**), similar to the non-treatment control (1.67%), MIDO- (0.43%) and SORA- (0.8%) treated cultures. However, there were still many clusters in QUIZ-treated blast cultures. FC analyses showed that there were 20.7% viable CD44+pBAD+ cells in the QUIZ-treated group (indicated by a red arrow, **Figure 1B**), suggesting the blast recovery process from the QUIZ-treatment was still ongoing. Finally, after 28 days after TKI treatment, we found that all culture of TKIs-treated MV4-11 recovered like non-treatment controls (**Figure 1C**). Also, our data suggests that both MV4-11 and in MOLM-14, another FLT3-mutated AML cell line, have similar phenotypes (blast regrowth) and molecular characterizations (increased pBAD) existing after TKI-treatment during the time-course studies (**Supplementary Figures 1C, D**). In summary, our time-course data (**Figure 1D**) suggests that CD44+pBAD+ is a biomarker for early TKI-resistant blasts, whose expression will downregulate after blasts have survived the TKI treatment.

### TKI-treatment upregulated PIM2/PIM3 in surviving blasts to protect against apoptosis

**Figure 2:**
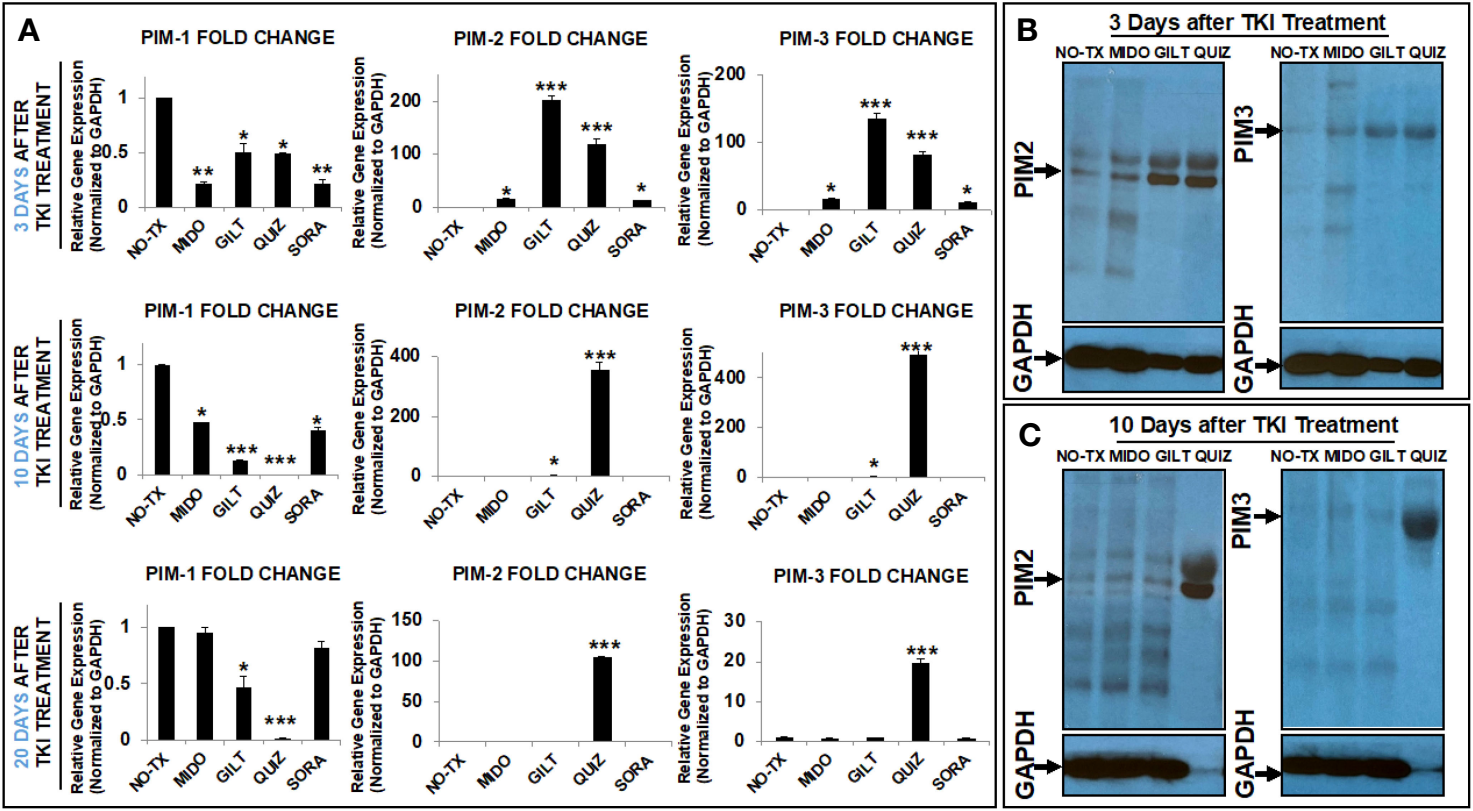
TKIs upregulated expression of PIM2 and PIM3 in TKI-treated MV4-11 *in vitro.* **(A)** 3, 10 and 20 days after TKI-treatment *in vitro*, MV4-11 cells of different experimental groups were collected for RNA isolation as described in Materials and Methods. The gene expressions of PIM1, PIM2 and PIM3 were analyzed by qPCR. Data of mRNA expressions show the fold change (normalized to GAPDH) in different experimental groups; **(B)** 3 days after TKI-treatment, the protein expressions of PIM2 and PIM3 in different experimental groups were analyzed by western blot; **(C)** 10 days after TKI-treatment, the protein expressions of PIM2 and PIM3 in different experimental groups were analyzed by western blot; Where applicable, data are means ± SEM and were analyzed by student “t” test. The significance of each experimental group was based on the NO-TX control group. *P<0.05, **P<0.01, ***P<0.005, N=3.

Next, to understand which kinase was responsible for the phosphorylation of BAD protein in TKI-resistant blasts, we screened the gene expression changes of all kinase candidates previously reported to phosphorylate BAD under physiological conditions (18). To follow time-course changes of kinases such as the PIM family in TKI-treated blasts, we also performed detailed qPCR and western blot analyses at different time intervals (3 days, 10 days, and 20 days). 3 days after TKI treatment, we found significant decreased expression of the PIM1 mRNA from all TKI-treated blasts when compared to the non-treatment control (NO-TX) (**Figure 2A**). On the contrary, PIM2 mRNA and PIM3 mRNA were significantly increased in all-TKIs-treated blasts when compared to the control (**Figure 2A**). In addition, our qPCR data showed that the increased folds of the PIM2 and PIM3 mRNA are the highest (203-fold up in PIM2 and 134-fold up in PIM3, **Figure 2A**) among all screened kinase genes (10-fold and 15-fold increase in AKT1 and PRKACA respectively, **Supplementary Figure 2**) in GILT-treated blasts. Further qPCR analyses revealed similar gene expression changes of PIM1/2/3 mRNA in QUIZ-treated blasts, with a significant decrease of 0.5-fold PIM1, increase of 120-fold PIM2, and increase of 81-fold PIM3 when compared non-treatment controls (**Figure 2A**). Next, western blots confirmed the significantly up-regulated PIM2/3 proteins at this time point (**Figure 2B**). For QUIZ-treated blasts, qPCR analyses revealed the lowest expression of PIM1 mRNA and the highest gene expressions of PIM 2/3 mRNA (PIM2: 355-fold up and PIM3: 494-fold up) at 10 days after the treatment when compared to the non-treatment controls (**Figure 2A**). These gene expression changes of PIM family were consistent with microscopic/FC data showing the appearance of early new clusters with 93.1% pBAD+ in QUIZ-treated blasts (QUIZ image and plot, **Figure 1A**). Western blots also confirmed the highest expressions of PIM2/3 proteins in QUIZ-treated blasts at 10 days after the treatment (**Figure 2C**) when compared to controls and other treatment groups. 20 days after TKI treatment, we found the full recovery of PIM1 mRNA in both MIDO- and SORA-treated blasts, normal expression of PIM2/3 in GLIT-treated blasts and moderate increase of PIM2/3 expressions in QUIZ-treated blasts (lower panel, **Figure 2A**). In summary, our data is consistent with previous reports that elevated PIM2 and PIM3 were responsible for TKI-resistance by protecting blasts against spontaneous apoptosis through phosphorylating BAD, leading to the poor survival of AML patients (31, 32).

### TKI-treated blasts activated JAK/STAT compensation pathways to promote signal transduction

**Figure 3:**
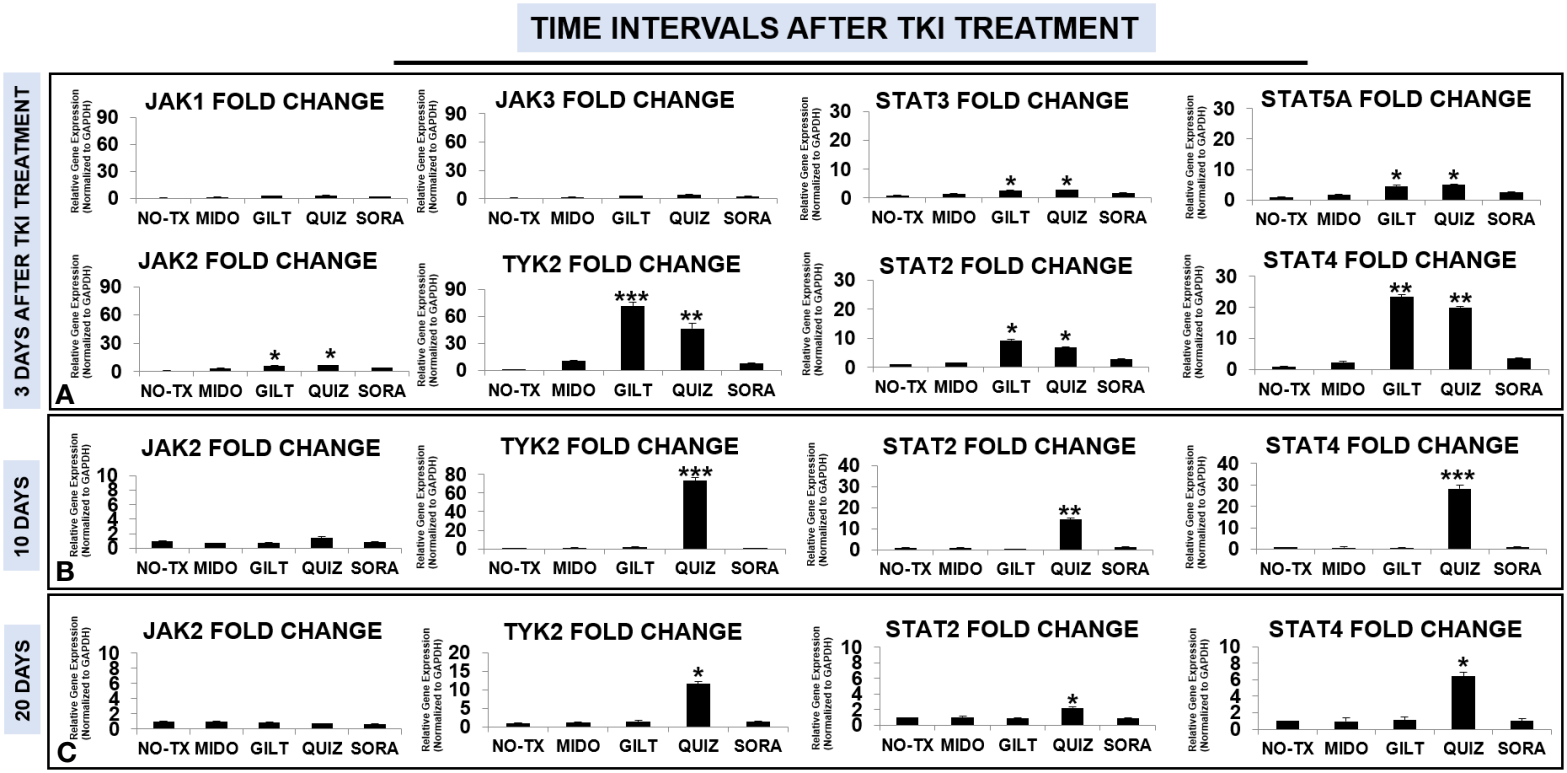
Gene expression changes of JAK/STAT signaling pathways in TKI-treated MV4-11 *in vitro*. 3 days **(A)**, 10 days **(B)**, and 20 days **(C)** after TKI-treatment in vitro, MV4-11 cells of different experimental groups were collected for RNA isolation and qPCR analyses of gene expression changes of JAK/STAT family members. Data of mRNA expressions show the fold change (normalized to GAPDH) in different treatment groups; Where applicable, data are means ± SEM and were analyzed by student “t” test. The significance of each experimental group was based on the NO-TX control group. *P<0.05, **P<0.01, ***P<0.005, N=3.

Enhanced activities of PIM2 have been reported in AML patients; however, the exact signaling pathway to activate PIM2 remains to be discovered in TKI-resistant blasts (31). The Janus kinase (JAK)/signal transducer and activator of transcription (STAT) pathway consist of a group of receptors and transmembrane proteins that recognize specific cytokines, essential for healthy hematopoiesis (33); in contrast, dysregulation of the JAK/STAT signaling pathway provides a survival advantage to leukemia cells by transmitting anti-apoptotic and proliferative signals (34). The sustained activation of FLT3-STAT5 signaling pathways was also found in FLT3-mut AML (35). To illustrate the signal transduction pathways for PIM2/3 activation in TKI-treated blasts, we performed qPCR screening of all gene expressions in the JAK/STAT pathway at different time intervals (3 days, 10 days, 20 days, and 28 days) after TKI exposure. 3 days after TKI treatments, our qPCR data displayed a significant increase of TYK2 mRNA (71-fold up) and JAK2 mRNA (6-fold up) in GILT-treated blasts when compared to non-treatment controls (**Figure 3A**). Similar increases of TYK2 mRNA (46-fold up) and JAK2 mRNA (6-fold up) occurred in QUIZ-treated blasts when compared to non-treatment controls (**Figure 3A**). In addition, the increased folds of both STAT4 mRNA and STAT2 mRNA were the highest among all screened STAT genes (23-fold up in STAT4 and 9-fold up in STAT2 versus 4-fold up in STAT5A and 2-fold up in STAT3, **Figure 3A**) in GILT-treated blasts when compared to non-treatment controls. Similar increases of STAT4 mRNA (19-fold up) and STAT2 mRNA (6-fold up) also occurred in QUIZ-treated blasts when compared to non-treatment controls (**Figure 3A**). 10 days after TKI treatment, qPCR analyses revealed the higher gene expressions of TYK2 mRNA (73-fold up), STAT2 mRNA (14-fold up), and STAT4 mRNA (28-fold up) in QUIZ-treated blasts when compared to non-treatment controls (**Figure 3B**); however, there was a gradual reduction trend of TYK2, STAT2 and STAT4 mRNA in QUIZ-treated blasts at 20 days after TKI treatment (**Figure 3C**). Finally, 28 days after TKI treatment, all TKIs-treated MV4-11 displayed normal gene expression of JAK/STAT families like non-treatment controls (not shown). Our data showing significant increase in STAT4 is consistent with a previous report that STAT4 signaling pathways regulated the process of PIM2-phosphorylation of BAD in chronic myeloid leukemia (CML) (36). Also, STAT4 activation was reported to involve STAT2 recruitment (37).

### TKI-treated blasts regained intrinsic homeostasis by upregulation of inhibitory pathways

**Figure 4:**
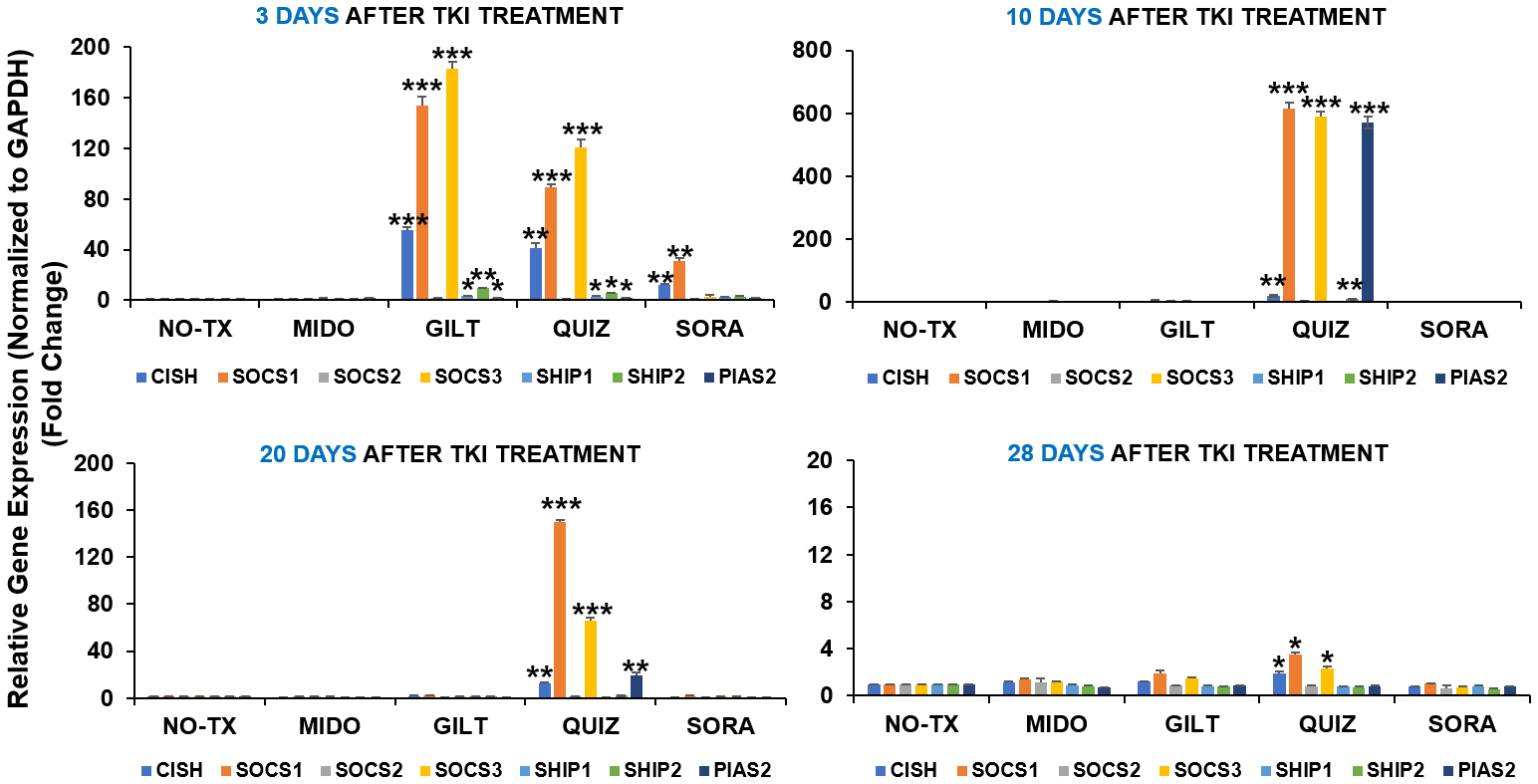
Gene expression changes of inhibitory pathways in TKIs-treated MV4-11 in vitro. 3 days, 10 days, 20 days and 28 days after TKI-treatment in vitro, MV4-11 cells of different experimental groups were collected for RNA isolation and qPCR analyses of gene expression changes of inhibitory pathway members. Data of mRNA expressions show the fold change (normalized to GAPDH) in different treatment groups; Where applicable, data are means ± SEM and were analyzed by student “t” test. The significance of each experimental group was based on the NO-TX control group. *P<0.05, **P<0.01, ***P<0.005, N=3.

Our time-course data revealing significant intracellular adaptation in TKI-treated blasts prompted us to question what happens to the intrinsic homeostasis of the blast, particularly how activated compensation pathways including TYK2/STAT4 signal transduction and PIM2/3 phosphorylation are switching off. Previously, JAK/STAT signaling pathway was reported to be tightly controlled by a number of endogenous feedback inhibitors, including suppressors of cytokine signaling (SOCS), protein inhibitors of activated STATs (PIASs), and protein tyrosine phosphatases (PTPs) like SHP1/2 (38). Dysregulation of inhibitory SOCS pathways were shown to confer resistance to FLT3 inhibitors (39). In this regard, we performed qPCR screening and detailed time-course analyses of all gene expressions in the SOCS family (40) at different time intervals (3 days, 10 days, 20 days, and 28 days) after TKI exposure. 3 days after TKI treatment, our qPCR data showed a significant increase of SOCS1 mRNA (153-fold up) and SOCS3 mRNA (182-fold up) in GILT-treated blasts when compared to non-treatment controls (Upper panel, **Figure 4**). Similar increases of SOCS1 mRNA (88-fold up) and SOCS3 mRNA (120-fold up) occurred in Quiz-treated blasts when compared to non-treatment controls (Upper panel, **Figure 4**). In addition, our qPCR data revealed significant increase of genes encoding phosphatases such as SHP1 mRNA (4-fold up), SHP2 mRNA (9-fold up) and Protein inhibitor of activated STAT2 (PIAS2, ~2-fold up) mRNA, reported as a negative regulator of STAT4 (41), in GILT-treated blasts when compared to non-treatment controls (Upper panel, **Figure 4**). Similar increase of SHP1 mRNA (3-fold up), SHP2 mRNA (5-fold up) and PIAS2 mRNA (~2-fold up) also occurred in QUIZ-treated MV4-11 blasts when compared to non-treatment controls (Upper panel, **Figure 4**). Remarkably, 10 days after TKI treatment, qPCR data revealed the significantly higher gene expression of SOCS1 mRNA (615-fold up), SOCS3 mRNA (590-fold up), SHP2 mRNA (9-fold up), and PIAS2 mRNA (570-fold up) in QUIZ-resistant blasts when compared to the non-treatment controls (**Figure 4**). At this moment, most surviving QUIZ-treated blasts were CD44+pBAD+ (93.1%, indicated by red arrow, QUIZ plot of **Figure 1A**), who formed new clusters (red arrows, QUIZ image of **Figure 1A**). 20 days and 28 days after TKI treatment, only Quiz-treated blasts still had a significant increase of CISH mRNA, SOCS1 mRNA and SOCS3 mRNA when compared to the non-treatment controls, but there was a trend of reduced expression with time (lower panel, **Figure 4**). In summary, our data suggests that multiple inhibitory pathways could be activated intracellularly during the recovery course of TKI-treated blasts.

### TKI-based combination therapies displayed superior efficacy in treating FLT3-mut AML *in vitro*

**Figure 5:**
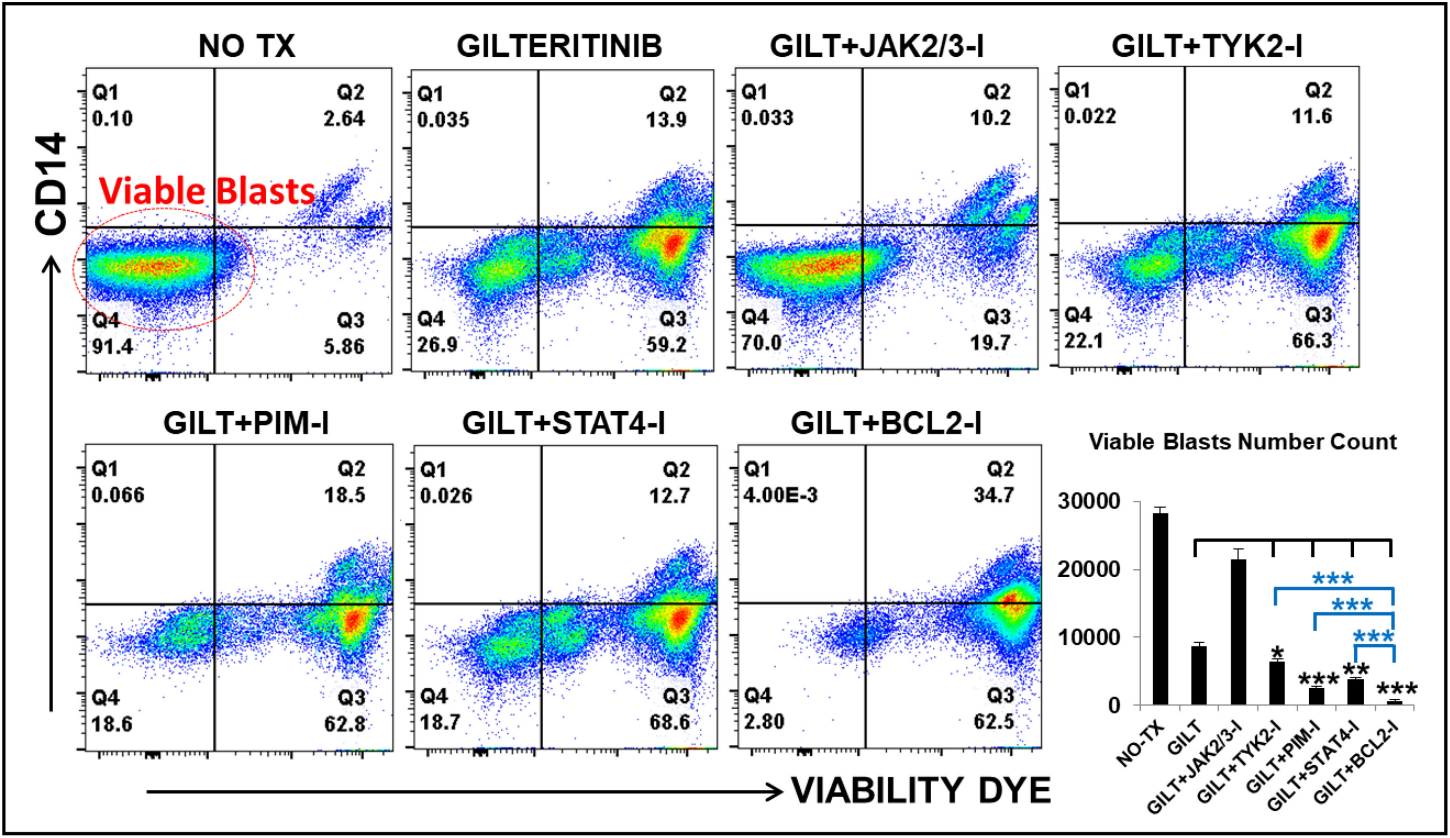
Gilteritinib-based combination treatments effectively treat MV4-11 *in vitro.* MV4-11 blasts were treated with Gilteritinib-based combination therapies for 3 days *in vitro*, and then treated cells were collected for flow cytometry (FC) analyses. Representative FC plots of different experimental groups with non-treatment (NO TX), 80nM GILTERITINIB (GILT), GILT+500nM JAK2/3-Inhibitor (I), GILT+500nM TYK2-I, GILT+100nM PIM-I, GILT+10µM STAT4-I and GILT+100nM BCL2-I; Red circle indicates the viable blasts; Lower right panel: Cumulative number count of viable MV4-11 blasts in different treatment groups; Where applicable, data are means ± SEM and were analyzed by ANOVA and student “t” test. The significance of each experimental group was based on the GILT-treatment alone or GILT+BCL2-I experimental groups. *p < 0.05, **P<0.01, ***P<0.005, N=3.

Based on detailed time-course studies of the survival mechanism *in vitro*, we found several potential therapeutic targets responsible for TKI-resistance. To determine the best treatment to prevent blast relapse, we explored different combinations of Gilteritinib (GILT) with pathway-driven small molecule inhibitors to examine whether they could overcome TKI-resistance *in vitro*. The detailed description of commercially acquired inhibitors targeting JAK2/3, TYK2, PIM1/2/3, STAT4 and BCL2 based on the forementioned mechanistic results, including their doses and catalogs, etc., can be found in **Materials and Methods**. Our FC analyses revealed significantly decreased numbers of viable blasts in GILT+BCL2-I (682), GILT+PIM-I (2481), GILT+STAT4-I (3881) and GILT+TYK2-I (6507) experimental groups, when compared to other treatments such as GILT alone (8664), GILT+JAK-I (21384) and non-treatment control (28326) (**Figure 5**). Among different combination treatment groups, GILT+BCL2-I was found to be the most effective therapy to reduce blasts *in vitro*. Our data suggest that the supplementation of TYK2/STAT4/PIM pathway-driven small molecule inhibitors to TKI treatments could be a novel therapeutic approach to treat AML and prevent disease relapse.

## Discussion

**Figure 6:**
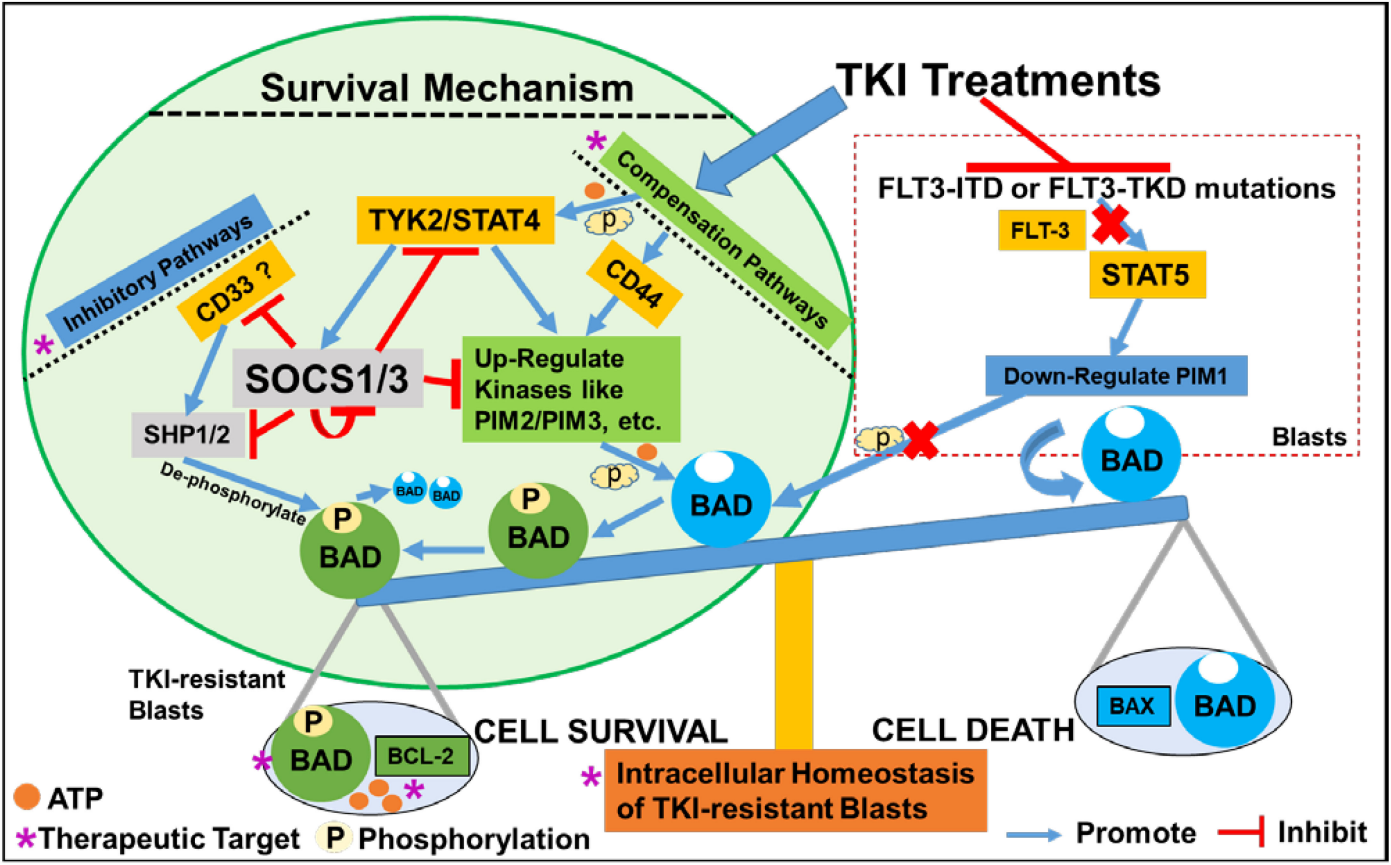
Schematic diagram illustrating the survival mechanism of TKI-resistant CD44+pBAD+ blasts *in vitro.* The TKI-treatments inhibit the FLT3-related down-stream signaling pathways such as STAT5-PIM1 axis known to phosphorylate BAD. Consequentially, non-phosphorylated BAD binds to BCL-2 and prevents BCL-2’s anti-apoptotic role, leading to the cell death of AML blasts. To survive, TKI-resistant blasts activate the compensation pathways such as non-classical JAK/STAT signaling or CD44 pathways to upregulate other PIM family kinases such as PIM2/PIM3 to phosphorylate BAD. The phosphorylated BAD (pBAD) fails to bind BCL-2 and BCL-2 will perform anti-apoptotic function, leading to the survival of TKI-resistant blasts. When blasts survive the TKI-treatments and their classical signaling pathways start to recover, blasts will upregulate inhibitory pathways to inhibit the activated compensation pathways to regain the intracellular and systemic homeostasis (42, 43). For example, CD33 recruits SHP1/SHP2 to de-phosphorylate pBAD towards BAD-associated cell death. TYK2/STAT4 activates SOCS1/SOCS3 inhibitory pathways to suppress SHP1/2, CD33, other active kinases, and even themselves to maintain homeostasis in TKI-resistant blasts *in vitro*.

In this study, we found that TKI-treatments could activate a JAK/STAT-PIM2/3 axis-mediated compensation pathway to support the relapse of a group of CD44+pBAD+blasts *in vitro* (**Figure 6**). Surviving blasts also underwent intracellular reprogramming to regain intrinsic homeostasis. Finally, we found that the supplementation of JAK/STAT-PIM pathways-driven inhibitors to TKI-treatments could effectively treat FLT3-mut AML *in vitro*.

CD44high+ populations are highly tumorigenic stem cells in breast cancer (44–46). Mechanistically, CD44 and its variant isoforms can activate different downstream signaling pathways to promote cancer cell invasion and proliferation (47). Furthermore, blocking hyaluronic acid, the main CD44 ligand, was found to inhibit the phosphorylation of BAD (48). In this study, our time-course FC analyses of QUIZ-resistant blasts showed the majority of the 94.2% pBAD+ cells had the same CD44 expression level as control or other treatment groups at 10 days after QUIZ-treatment (**Figure 1A**); however, at 20 days after QUIZ-treatment, some surviving QUIZ-resistant blasts displayed reduced pBAD+ but higher CD44+ expression (**Figure 1B**). The same phenomena could be observed in some GILT-resistant blasts showing pBAD-/CD44high+ (**Figure 1A**). Previously, higher expressions of CD44 were found to adhere immature blasts to the bone marrow matrix, resulting in their failed differentiation and sustained clonal proliferation in AML patients (49). It’s possible that instead of phosphorylating BAD, increased CD44high might function as an adhesion molecule to assist the proliferation of survived blasts.

### TKI-resistant blasts require homeostatic adaptation to survive and relapse *in vitro* (**Figure 6**)

Homeostasis is essential for the maintenance of human health and healthy aging at both cellular (42) and systemic levels (43). Adjustable genetic and proteomic networks to maintain intracellular homeostasis also have been proposed (50, 51), some of which were experimentally explored (42, 52–55). As part of an ancient evolutionary mechanism of robustness for all living beings, redundant elements can compensate for the loss/lesion of another family gene, such as the activation of PIM2 to compensate for the lack of PIM1 to maintain homeostasis (56); however, this compensation pathway could be an obstacle for effective cancer treatments (57). In this regard, to overcome the TKI-resistance, it’s essential to discover the signaling pathways responsible for the activation of compensatory PIM2/3. The JAK/STAT signaling pathway is composed of ligand-receptor complexes that are activated by various pro-inflammatory cytokines regulated by NFKB family (33). IL-12, a cytokine activating TYK2/STAT4 pathways was reported be regulated by non-canonical NFKB pathway (58, 59). Previously, we found that TKI-treatment could activate a non-canonical NFKB2/Cytokines/Chemokines/CXCR2 pro-leukemia inflammatory pathway to initiate the survival and relapse of FLT3-mut AML blasts *ex vivo* (60). Consistent with above reports, our time-course analyses revealed that TKI-treatment could also activate TYK2/STAT4 signaling pathways (73-fold up/28-fold up in QUIZ-treated blasts, **Figure 3B**). Recently, STAT4-mediated TKI-resistant pathways were found to compensate for the functional loss of STAT5 (TKI-sensitive) and activate PIM2 to restore the phosphorylation of BAD to support CML relapse (36). PIM2 has been reported to be a downstream targeted gene of STAT4 (61). Thus, it’s possible that cytokines-induced JAK/STAT and CD44 pathways work together to activate PIM2/3 to phosphorylate BAD to initiate the survival of TKI-treated blasts and then blast relapse *in vitro* (**Figure 6**).

Many negative regulators are involved to maintain the balance of JAK/STAT signal transduction pathway (33). CD33, a well-known blast biomarker, was reported to be a myeloid-specific inhibitory receptor containing a cytoplasmic immunoreceptor tyrosine-based inhibitory motif (ITIM) with functions in recruiting the phosphatases SHP-1 and SHP-2 (62, 63), which might in turn to de-phosphorylate pBAD in TKI-resistant blasts towards BAD-associated cell death (**Figure 6**). The rapid induction of suppressors of cytokine signaling (SOCS) proteins depends on STAT activation, and the duration/intensity of JAK/STAT pathway responses (64). SOCS1/3 are negative regulators of the IL-6/STAT3/NFKB axis, whose dysfunction leads to various cancers (65). Interestingly, SOCS3 was reported to not only compete with SHP1/SHP2 by binding to ITIMs of CD33 but also accelerating proteasomal degradation of itself and CD33 (66). STAT4 pathways were reported to regulate the activation of SOCS1/SOCS3 (61). Thus, SOCS1/3 inhibitory pathways could be quickly upregulated upon the activation of JAK/STAT-PIM2/3 axis to shut down redundant signaling pathways and regain intrinsic homeostasis in blasts that survive the treatment (**Figure 6**).

Finally, based on our mechanistic studies, we proposed multiple essential pathways needed to be therapeutically targeted to overcome TKIs-resistance (indicated by purple stars, **Figure 6**). To verify our hypothesis, we explored several therapies combining GILT with commercially acquired inhibitors targeting the central TYK2/STAT4-PIM2/3 compensation pathway to treat MV4-11 blasts *in vitro*. The combination of GILT with Venetoclax, a BCL-2 inhibitor (I), or AZD1208, a PIM-I or (R)-Lisofylline, a STAT4-I, or PF-06826647, a TYK2-I was demonstrated to have better therapeutic effect than GILT alone *in vitro*. However, the combination of GILT+JAK-I (AT9283) failed to display additive efficacy suggesting that AT9283 might need additional dose adjustment for optimal therapies based on data from a previous report showing its dose and time-dependent manner of inhibiting cancer cell lines (67). The current study has limitations including: 1) The survived blasts might undergo transient resistance to the TKI-treatment because of one-dose TKI-treatment for a short-term in this study (Please see details in Materials and Methods). Thus, duration effects with multiple doses of TKI treatments should be evaluated in the future studies. 2) All analytic approaches (qPCR, WB, drug combination treatments) should be performed in more AML cell lines, with the best scenario in primary patient samples with detailed transcriptome analyses (single-cell or bulk) to broaden the relevance of this study; 3) The activation of JAK/STAT signaling pathways and inhibitory pathways was based on mRNA expression, which warrants further investigation at the protein analytic level; 4) These promising *in vitro* results should be evaluated for both therapeutic efficacy and potential toxicity in AML patient-derived xenograft murine models *in vivo*.

### Importance of dissecting treatments-activated compensation pathways in cancer medicine

AML continues to be a challenge to treat since the introduction of cytarabine/daunorubicin (7 + 3) in 1973 (50 years lapsed) (68). Disease relapse is common with a mysterious 2^nd^ wave of relapsed leukemia occurring in remission patients (69). The hallmarks of cancer cells have been brilliantly organized to provide a principle framework of their tumorigenic capabilities under physiological condition (70); however understanding how cancer cells perform intracellular homeostatic reprogramming to survive the treatments and adapt to their new microenvironment is also crucial to the development of effective cancer medicines. Besides a novel JAK/STAT-PIM2/3 axis-mediated compensation pathway discovered in this study, accumulating evidence (our previous studies and others) also suggests that non-canonical NFKB2 pro-leukemia inflammatory pathways could play an essential role in treatment-resistance in both standard treatments and targeted therapies for AML (60, 71). In this regard, recognition of this applicable concept of treatments-activated compensation in cancer field, and acquisition of a comprehensive database of compensatory gene/protein repertoires in treated neoplasia might provide new therapeutic means to treat human cancer effectively.

In conclusion, we provide the first evidence that CD44+pBAD+cells require intracellular homeostatic adaptation to promote blast relapse *in vitro*. Inhibition of TKI-activated compensation pathways and blast homeostasis could potentially be a novel therapeutic strategy to treat FLT3-mut AML and prevent disease relapse *in vivo*.

## Data availability statement

The original contributions presented in the study are included in the article/**Supplementary Material**. Further inquiries can be directed to the corresponding author.

## Ethics statement

Ethical approval was not required for the studies on humans in accordance with the local legislation and institutional requirements because only commercially available established cell lines were used.

## Author contributions

YX: Conceptualization, Data curation, Formal Analysis, Funding acquisition, Investigation, Methodology, Project administration, Resources, Software, Supervision, Validation, Visualization, Writing – original draft, Writing – review & editing. DB: Writing – review & editing. C-SC: Writing – review & editing. LT: Writing – review & editing. JX: Investigation, Writing – review & editing. BP: Investigation, Writing – review & editing. IV: Investigation, Writing – review & editing. MR: Writing – review & editing. HC: Funding acquisition, Investigation, Supervision, Writing – review & editing.
