# Supplementary material for: Transient TKI-resistant CD44+pBAD+ blasts undergo intrinsic homeostatic adaptation to promote the survival of acute myeloid leukemia in vitro
Source: Front Oncol. 2023 Nov 8;13:1286863. doi: 10.3389/fonc.2023.1286863 (PMC10664142; doi:10.3389/fonc.2023.1286863)
Supplement: Supplementary file 1 [file DataSheet_1.pdf]

Transient TKI-Resistant CD44+pBAD+ Blasts Undergo Intrinsic Homeostatic Adaptation To Promote The Survival of Acute Myeloid Leukemia *In Vitro*

Authors:

Yi Xu[1, 2, 3@], David J. Baylink[2, 3], Chien-Shing Chen[1, 3], Laren Tan[3, 4], Jeffrey Xiao[2], Brandon Park[2], Ismael Valladares[2], Mark E Reeves[1, 3], Huynh Cao[1, 3]

Affiliations:

[1]Division of Hematology and Oncology, Loma Linda University Medical Center and Loma Linda University Cancer Center, Loma Linda University Health, Loma Linda, California.

[2]Division of Regenerative Medicine, Department of Medicine, Loma Linda University, Loma Linda, California.

[3]Department of Medicine, Loma Linda University School of Medicine, Loma Linda University Health, Loma Linda, California.

[4]Department of Pulmonary, Critical Care, Hyperbaric and Sleep Medicine, Loma Linda University Medical Center, Loma Linda, California.

Supplementary Table 1: List of reagents used in this study.

List of Reagents				
**Antibody/Reagents**	**Abbreviation/Name in the text**	**Cat. #**	**Company**	**Species Reactivity**
**CD14-APC**	CD14	301812	Biolegend	Human
**Viability Dye eFluor™ 780**	**Viability Dye**	65-0865-14	eBioscience	
**CD33-PERCP**	CD33	341640	BD	Human
**CD44-PE/Cyanine7**	CD44	338816	Biolegend	Human
**pBAD-FITC**	pBAD	SC-166932	SANTA CRUZ	Human
**Ki67-PE**	Ki67	350503	Biolegend	Human
**Midostaurin**	MIDO	M1323	Sigma Aldrich	
**Gilteritinib (ASP2215)**	GILT	S7754	SELLECKCHEM	
**Quizartinib**	QUIZ	A10027	ADOOQ	

**Sorafenib**	SORA	SML2633	Sigma Aldrich	
**Venetoclax (ABT-199)**	BCL2-I	A12500	ADOOQ	
**PF-06826647**	TYK2-I	HY-126290	MedChemExpress	
**AT9283**	JAK2/3-I	HY-50514	MedChemExpress	
**(R)-Lisofylline**	STAT4-I	HY-109854A	MedChemExpress	
**AZD 1208**	PIM-I	20235	Cayman	

Supplementary Table 2: List of primers (OriGene) used in this study.

#	Name (HUMAN)	Forward Sequence	Reverse Sequence
1	**PIM1**	CGAGCATGACGAAGAGATCAT	TCGAAGGTTGGCCTATCTGA
2	**PIM2**	GGCAGCCAGCATATGGG	TAATCCGCCGGTGCCTGG
3	**PIM3**	GGACAAGGAGAGCTTCGAGAAG	CTCCTTCACCACGTGCTTCACA
4	**JAK1**	GAGACAGGTCTCCCACAAACAC	GTGGTAAGGACATCGCTTTTCCG
5	**JAK2**	CCAGATGGAAACTGTTCGCTCAG	GAGGTTGGTACATCAGAAACACC
6	**JAK3**	AGTGACCCTCACTTCCTGCTGT	GGCTGAACCAAGGATGATGTGG
7	**TYK2**	GGTTGACCAGAAGGAGATCACC	TCCTCGTCATCCATCTTGCCCT
8	**STAT2**	CAGGTCACAGAGTTGCTACAGC	CGGTGAACTTGCTGCCAGTCTT
9	**STAT3**	CTTTGAGACCGAGGTGTATCACC	GGTCAGCATGTTGTACCACAGG
10	**STAT4**	CAGTGAAAGCCATCTCGGAGGA	TGTAGTCTCGCAGGATGTCAGC
11	**STAT5A**	GTTCAGTGTTGGCAGCAATGAGC	AGCACAGTAGCCGTGGCATTGT
12	**CISH**	GCATAGCCAAGACCTTCTCCTAC	ACGTGCCTTCTGGCATCTTCTG
13	**SOCS1**	TTCGCCCTTAGCGTGAAGATGG	TAGTGCTCCAGCAGCTCGAAGA

14	**SOCS2**	GGTCGGCGGAGGAGCCATCC	GAAAGTTCCTTCTGGTGCCTCTT
15	**SOCS3**	CATCTCTGTCGGAAGACCGTCA	GCATCGTACTGGTCCAGGAACT
16	**PIAS2**	GTTCTTGGTGTCCAATGAGACCG	TGCTTGCCTCACTGGCTACAGT
17	**SHP1**	TTGACCACAGCCGAGTGATCCT	CTGGCGATGTAGGTCTTAGCGT
18	**SHP2 (PTPN11)**	GACTTTTGGCGGATGGTGTTCC	CGGCGCTTTCTTTGACGTTCCT
19	**GAPDH**	GTCTCCTCTGACTTCAACAGCG	ACCACCCTGTTGCTGTAGCCAA
20	**AKT1**	TGGACTACCTGCACTCGGAGAA	GTGCCGCAAAAGGTCTTCATGG
21	**PRKACA**	CCACTATGCCATGAAGATCCTCG	CGAGTTTGACGAGGAACGGAAAG

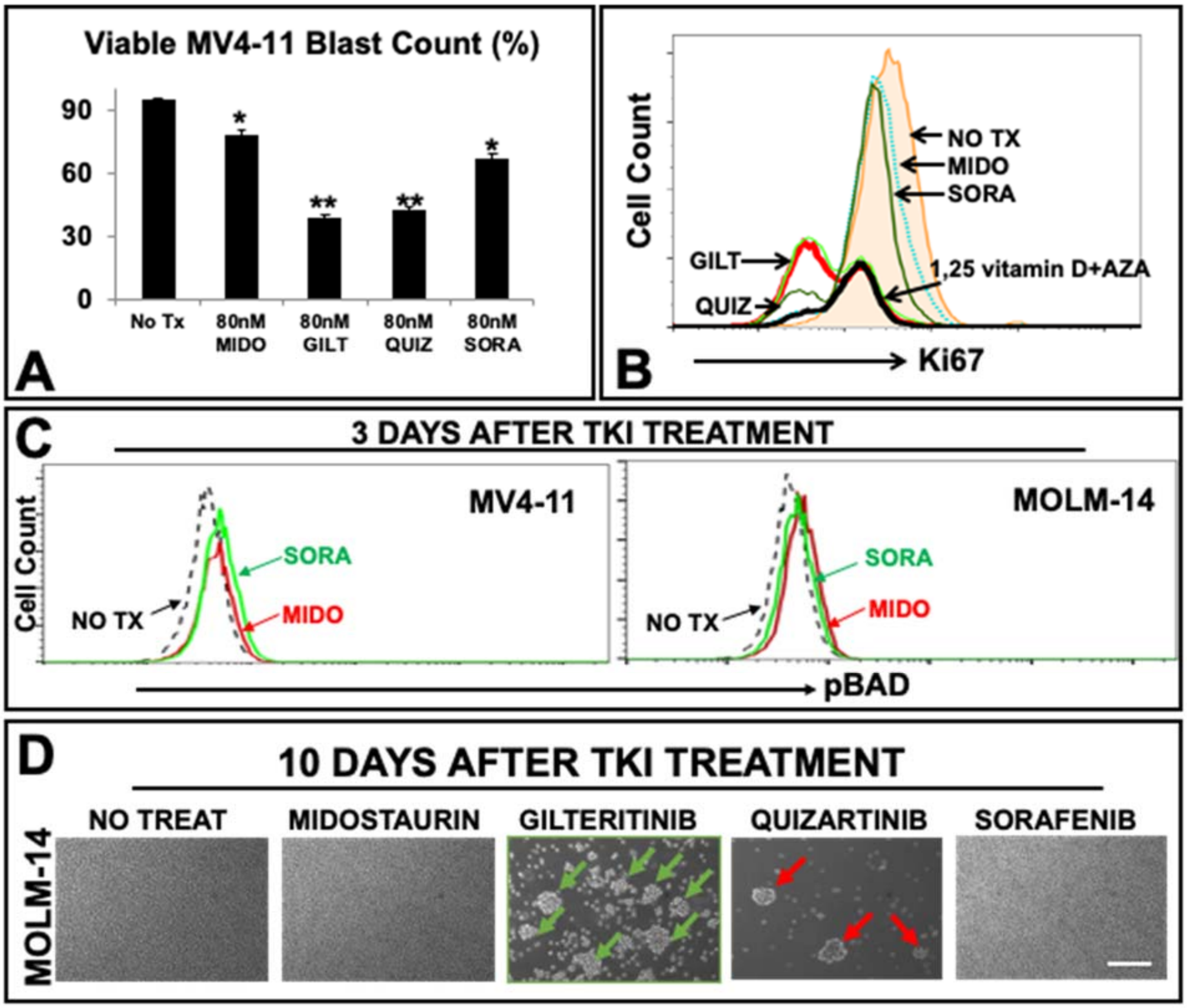

Supplementary Figure 1. Therapeutic effect of different TKIs on MV4-11 and MOLM-14 Cells *In Vitro*

A) Cumulative flow cytometry (FC) percentage data of viable MV4-11 blasts after 3-day treatment *in vitro*;

B) Representative FC histogram of Ki67 expression in different treatment groups of MV4-11 blasts after 3-day treatment *in vitro;* A treatment group of 1,25vitamin D (Sigma Aldrich H-089-1ml)+AZA (5-Azacitidine, Celgene) was studied as a therapeutic control, which was previously reported by our group to effectively reduce the proliferation of FLT3-mutated blasts *in vitro* (1);

NO Treatment (NO TX): Ki67+ mean fluorescence intensity (MFI): 384; MIDO (Ki67+MFI: 227); SORA (Ki67+MFI: 200); GILT (Ki67+MFI: 81); QUIZ (Ki67+MFI: 60); 1,25vitamin D+AZA (Ki67+MFI: 116);

C) Representative FC histograms of pBAD expression in MIDO- and SORA- treatment groups of MV4-11 and MOLM-14 cells after 3-day treatment *in vitro*;

D) Representative phase-bright images of different experimental groups of MOLM-14 blasts after 10 days of TKI-treatment *in vitro;* Scale bar: 500 µm; Green arrows indicate re-grown clusters in Gilteritinb (GILT)-treated MOLM-14 cells; Red arrows indicate re-grown clusters in Quizartinib (QUIZ)-treated MOLM-14 cells;

Our data of **C)** and **D)** demonstrated that there were increased pBAD expressions in both MIDO- and SORA-treatment groups of MV4-11 and MOLM-14 cells after 3-day treatment, occurring before MIDO- or SORA-treated blasts recovered like non-treated blasts after 10 days *in vitro* (3-10 days' recovery for MIDO/SORA-treated blasts). Similar phenotypes and increased pBAD expressions were also found in delayed relapse in GILT-treated blasts (10-20 days' recovery for GILT-treated blasts) and QUIZ-treated blasts (20-28 days' recovery for QUIZ-treated blasts) (**Fig.1A and 1B**).

Where applicable, data are means ± SEM and were analyzed by student "t" test. The significance of each experimental group was based on the NO-TX control group. *$p < 0.05$, **$P<0.01$, N=3.

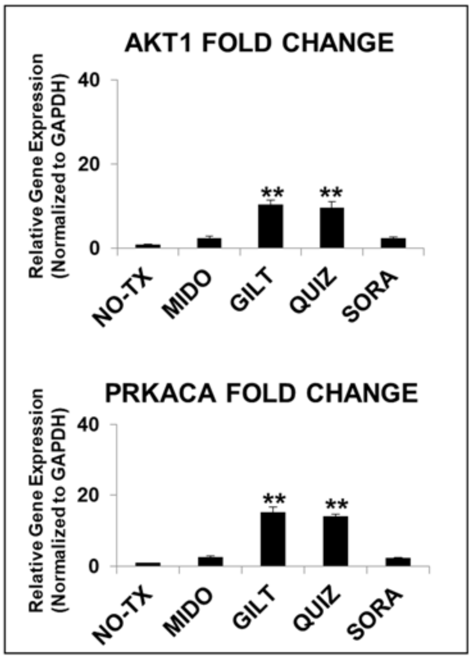

Supplementary Figure 2. qPCR analyses of Gene Expression Changes of AKT1 and PPKACA in TKI-treated MV4-11

3 days after TKI-treatment *in vitro*, MV4-11 cells of different experimental groups were collected for RNA isolation as described in Materials and Methods. The gene expressions of AKT1 and PPKACA were analyzed by qPCR. Data of mRNA expressions show the fold change (normalized to GAPDH) in different experimental groups;

Where applicable, data are means ± SEM and were analyzed by student "t" test. The significance of each experimental group was based on the NO-TX control group. **P<0.01, N=3.

1. Xu, Y., Payne, K., Pham, L. H. G., Eunwoo, P., Xiao, J., Chi, D., Lyu, J., Campion, R., Wasnik, S., Jeong, I. S., Tang, X., Baylink, D. J., Chen, C. S., Reeves, M., Akhtari, M., Mirshahidi, S., Marcucci, G., and Cao, H. (2020) A novel vitamin D gene therapy for acute myeloid leukemia. *Transl Oncol* **13**, 100869
